# A Qualitative Exploratory Study of the Political Commitment for Nutrition Programming: A Case Study of Anambra and Kebbi States of Nigeria

**DOI:** 10.3390/ijerph21020175

**Published:** 2024-02-02

**Authors:** Oluchi Ezekannagha, Scott Drimie, Dieter Von Fintel, Busie Maziya-Dixon, Xikombiso Mbhenyane

**Affiliations:** 1 Division of Human Nutrition, Department of Global Health, Faculty of Medicine and Health Sciences, Tygerberg Campus, Stellenbosch University, Cape Town 8000, South Africa; 2 International Institute of Tropical Agriculture, Ibadan 200001, Nigeria; 3 CGIAR System Organization, c/o Alliance of Bioversity and CIAT, 00153 Rome, Italy; 4 Department of Economics, Faculty of Economic and Management Sciences, Stellenbosch University, Stellenbosch 7602, South Africa

**Keywords:** political commitment, nutrition programming, nutrition sensitivity, Anambra state, Kebbi state, Nigeria

## Abstract

In Nigeria, varying levels of malnutrition across states present a critical challenge to public health, demanding tailored policy responses. This paper delves into the specific issues and dynamics influencing nutrition programs in the country. Advocating for nutrition-sensitive actions requires analyzing context-specific political commitment. This article presents a case study on two Nigerian states with varying malnutrition profiles to explore the political economy of nutrition. The study used stakeholder analysis, in-depth interviews, and semi-structured interviews through workshops, incorporating the Political Commitment Rapid Assessment Tool. The objective was to measure political commitment, the window of opportunity for action, and stakeholder analysis. The results showed that despite having a significant child malnutrition problem, Kebbi State received a high political commitment to nutrition, with proportions ranging from 0.67 to 1 in each of the six domains measured. On the other hand, Anambra State, where malnutrition was less severe, had varying commitment levels. Institutional commitment was marginally high (0.67), expressed commitment was high (0.71), and budgetary commitment was lower at 0.33. Kebbi had better support for programs dependent on foreign donors than Anambra. Both states need to use media to increase awareness about nutrition issues. When the nutrition situation is severe, foreign donors’ influence grows. In conclusion, there are opportunities for strategic framing and advocacy of the nutrition profile of the states. Local state media can be effective, and institutional coordination committees that include various sectors already facilitate commitment to nutrition actions. However, individual, uncoordinated sectoral action can counterbalance the benefits of these committees. Further possibilities to generate political commitment for nutrition in the states are available. This study not only offers insights into the effectiveness of political strategies in addressing malnutrition but also lays the groundwork for future research and provides actionable recommendations for government policymaking.

## 1. Introduction

Political economy factors are crucial in determining the success of nutrition interventions, programs, and policies as they involve the collaboration of people, processes, and resources [1,2]. In this context, nutritional intervention refers to coordinated actions to improve nutrition outcomes. Addressing challenges to mainstream nutrition as a policy priority is crucial [3].

As Baker et al. [4] state, a systemic approach to nutrition involves the participation of multiple sectors, including environmental health, water, sanitation, and hygiene (WASH), and education, at various levels and with multiple stakeholders. The first step in addressing the problem is to recognize the need for more political commitment. It is essential to conduct political commitment analysis and stay aware of how nutrition issues are addressed across different spheres of influence. This is crucial for nutrition advocacy and to bring these issues to the policy discourse.

This definition of political commitment to nutrition based on sufficient evidence is “the intent and sustained actions over time by societal actors to achieve the objective of reducing and eliminating the manifestations and causes of malnutrition” [4]. Focusing only on political commitment to nutrition is insufficient when discussing malnutrition. This is because policymaking involves a formal exchange of ideas and values that go beyond just discussing malnutrition. Therefore, it is important to understand other aspects of political commitment to nutrition.

Nigeria has developed various nutrition-specific interventions in collaboration with foreign partner agencies under the guidance of the Federal Ministry of Health. Some of these programs include the Maternal Newborn and Child Health Week (MNCHW) held twice annually, the Saving One Million Lives Programme for Results (SOML-PforR), and the Community Health Influencers, Promoters, and Services (CHIPS) [5]. This study argues for innovative approaches to address malnutrition, including mainstreaming nutrition into nutrition-sensitive sectors such as agriculture, water, sanitation and hygiene, education, and social protection [6].

Political commitment towards nutrition can be measured through three indicators: budgetary, expressed, and institutional commitment. Budgetary commitment highlights how resources are allocated and mobilized for nutrition-related initiatives and strategic plans. Expressed commitment refers to the importance of nutrition in key state policies and programs. Institutional commitment involves the presence of a multisectoral nutrition body and the formulation of strategic plans and policies. Moreover, political commitment also depends on the level of coordination between different sectors and relevant government agencies [7]. Kingdon [8] believes that when society faces problems, potential policy solutions and political factors align, leading to a chance to advance policies on any agenda. The problem stream refers to the policy issues that a society needs to address.

In contrast, the policy stream includes the potential solutions to these problems proposed by policymakers, experts, and lobby groups. The politics stream refers to factors such as changes in government, legislative turnover, and fluctuations in public opinion. The convergence of these three streams creates an opportunity for exploring change, known as an open window of opportunity. This concept is based on Kingdon’s theory, which has been applied in various contexts and fields [9].

An institution’s success depends on leadership, governance, composition, and framing strategies [10]. As found in a study, the key elements required for generating political momentum to support maternal health include unity among policymakers, establishing sustainable institutions, societal awareness, and collaboration between national initiatives and civil society [11]. These factors also apply to nutrition and form the basis of this article [8,11].

Despite the availability of research on political commitment, existing studies have primarily focused on the national level, concentrating on establishing nutrition coordination bodies and intersectoral nutrition coordination [12]. However, challenges that emerge at the national and sub-national levels interfere with coordinating policy across government sectors and implementing nutrition actions, especially nutrition-sensitive ones [4]. Among these challenges is the fragmentation of policy frameworks, often seen at the national level, where competing priorities among different government ministries create barriers to unified action on nutrition. Budget constraints and resource limitations further complicate the prioritization of long-term nutrition strategies. At the sub-national or state level, issues such as disparities in resource allocation, varying degrees of political will, and diverse regional political dynamics significantly impact the execution of nutrition programs. One such challenge is the minimal sense of responsibility for nutrition outside the health sector, leading to weak horizontal coherence among sectors [13]. This is compounded by bureaucratic inertia and inadequate training of personnel in non-health sectors, making it even more challenging to find nutrition funding and effectively implement nutrition-sensitive interventions.

This study is highly relevant to exploring efforts to generate political commitment and identifying windows of opportunity for nutrition programming at the state level in Nigeria. Specifically, this research aims to identify and understand the windows of opportunity that can be leveraged for effective nutrition interventions. By examining the political, economic, and social factors influencing policymaking and implementation, this study aims to provide comprehensive insights into the strategies that can enhance the success of nutrition programs. Such an understanding is crucial for developing more effective and sustainable nutrition interventions, ultimately contributing to better health outcomes in the Nigerian context.

## 2. Materials and Methods

### 2.1. Study Design and Setting

The study utilized qualitative methods to assess political commitment to nutrition-sensitive interventions in Kebbi and Anambra States, Nigeria, using the Political Commitment Rapid Assessment Test (PCOM-RAT) in workshops and interviews [8]. The PCOM-RAT has been applied to assess national political commitment in India and Senegal [14,15,16]. The survey tool assesses political commitment to food security and opportunities for nutrition policy [15]. The PCOM-RAT has three main objectives. Firstly, to determine the present level of political backing for nutrition policies and gauge any recent changes in priority. Secondly, to pinpoint opportunities for the advancement of food and nutrition policies. Lastly, it will aid in formulating strategies to promote food and nutrition policies within a specific political framework [15].

### 2.2. Population and Sampling

There are spatial differences in under-5s stunting rates between the South and North of Nigeria. Kebbi, located in the North, has a high stunting rate of 66.1% [17], while Anambra State has a stunting rate of 14%, which is relatively low compared to other states in the country’s North. For the study, we purposely selected five ministries/sectors: health, agriculture, education, environment, and social welfare. These sectors have significant roles in domains that can have nutrition-sensitive impacts [18].

The data was collected between December 2017 and May 2018. The collection process involved conducting workshops and in-depth interviews with carefully selected officials. Ten officials were invited to participate in the workshops in each state, and if the senior stakeholder was unavailable, they nominated a substitute. The Ministry’s Permanent Secretary or Commissioner nominated the workshop participants, who were staff leading the Ministry’s nutrition section. Mid- and senior-level staff ranks such as permanent secretaries, directors, and State Nutrition Officers were purposively recruited for the in-depth interviews.

### 2.3. Public Involvement

In this study, the involvement of the stakeholders was pivotal. Their roles included providing firsthand experiences and perspectives on the political economy for nutrition in the states.

### 2.4. Data Collection Procedures

#### 2.4.1. The instrument

The PCOM-RAT [19] consists of factual and subjective questions. The approach has three main components. First, it assesses political commitment by gathering information on expressed, institutional, and budgetary support. Second, it investigates the policy window of opportunity by examining problems, policies, politics, and other external factors. Lastly, it analyzes stakeholders and institutions to identify supporters and opponents of nutrition in the different sectors.

#### 2.4.2. Data Collection Procedures

Data was collected using the PCOM-RAT during a one-day workshop or an interviewer-administered questionnaire. The lead researcher and an assistant facilitated the workshops in both states to ensure consistency in the study environment. The PCOM-RAT questions were administered using an agenda during the one-day workshop. Seven participants from Anambra and eight from Kebbi, representing varying levels of the Ministries, attended the workshop, as shown in Table 1. All participants were high or mid-level government staff and provided their answers jointly with discussions allowed before the agreement. The workshop was conducted in English and recorded for accurate information gathering and transcription.

In addition, three in-depth interviews were conducted in Anambra state, while two were conducted in Kebbi State with participants who could not attend the workshop. These interviewees were selected based on their availability, willingness to participate, and representative roles within the government structure. This approach was taken to obtain a diverse perspective from various levels of the ministries who could not attend the workshop. The PCOM-RAT was used as an interview guide to discuss political commitment to ensure that the information obtained was similar to the data gathered during the workshop discussions. The workshop discussions were conducted to ensure data saturation until no new themes emerged, indicating that the data collected was comprehensive and representative of the subject matter. Trustworthiness was established through methods such as member checking in the workshops, where participants reviewed and confirmed the accuracy of their responses, and triangulation, which involved comparing data from workshops with the interviews and/or available documents.

All participants were assured of confidentiality, and their data was anonymized during analysis. The participants were informed that the interviews and workshops were recorded, and their consent was obtained at the beginning of the workshops and interviews.

### 2.5. Data Analysis Approach

All recordings were transcribed using MAXQDA (VERBI GmbH) software version 12.3.1. The notes and audio files were then organized based on the workshop location. The answers to the PCOM-RAT questions were recorded in an Excel spreadsheet, scored, and depicted in radar plots. For objective questions, a score of zero or one was given, with “yes” receiving a score of one. Subjective questions were scaled from 1–10 and reclassified as binary (1 for scores of 7 or higher). Budgetary questions were scaled from 0–3, with three representing adequate resources.

The workshop discussions and interviews were transcribed and coded line by line [20]. After coding all transcripts, we used PCOM-RAT themes to analyze them. The PCOM-RAT data, workshop discussions, and key informant interviews were compared and interpreted.

In conducting the SWOT (strengths, weaknesses, opportunities, and threats) analysis, we engaged in qualitative analysis, reviewing governmental policies, health reports, and donor strategies to identify strengths and weaknesses within each state’s approach to nutrition. Opportunities and threats were determined by examining the broader socio-political and economic contexts, considering potential changes in donor support and policy focus. This methodical approach enabled us to develop a comprehensive SWOT analysis, highlighting key factors affecting nutritional outcomes in both states.

The interviewer was trained in qualitative research, had extensive experience in qualitative research, and was familiar with the subject matter conducted in the interviews. This ensured a high level of competency in eliciting in-depth responses from participants.

## 3. Results

**Figure 1:**
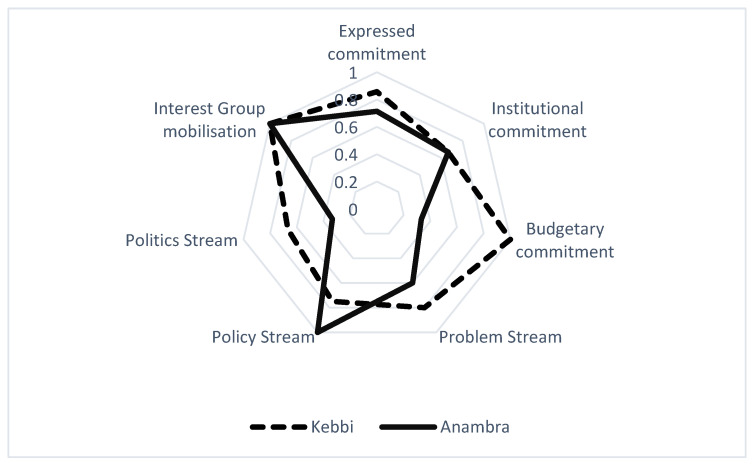
Proportions of political commitment in each theme.

As depicted in the radar chart below, Figure 1, we quantitatively represent the political commitment levels across various themes, with 0 reflecting an absence of commitment and 1 denoting comprehensive commitment. The gradations between these two extremes illustrate the degrees of commitment within each thematic area. This chart serves as an initial visual summary of the comparative political landscapes in Anambra and Kebbi states, setting the stage for a nuanced discussion on their respective commitment levels. Notably, both states demonstrated parallel performance in institutional commitment and interest group mobilization. However, a divergence is observed in other areas: Kebbi State exhibits a more pronounced commitment to budgetary aspects and addressing the problem and politics streams. In contrast, Anambra State’s higher scores in the policy stream underscore its focus on policy-oriented solutions. These variations, as systematically plotted in Figure 1, provide a foundational overview for the detailed analyses that follows.

The qualitative results of the political commitment analysis, interpretation, and discussion are presented under the subheadings below.

### 3.1. Political Commitment by States

#### 3.1.1. Expressed Commitments

Participants in both states used Maternal Newborn and Child Health Week (MNCHW) as a reference point for their responses, discussing their commitment to various aspects of nutrition in relation to this event. Administrators of three ministries in Kebbi State expressed a high level of commitment to nutrition, with the Ministry of Social Welfare focusing on Orphan and Vulnerable Children events, the Ministry of Environment advocating for WASH and health, and the Ministry of Health expressing commitment to MNCHW [21] In Anambra State, the Commissioner of the Ministry of Education mentioned nutrition with early marriage. Nutrition was also cited as a prominent topic raised by the Governor and his wife during the formal commencement of MNCHW [22].

#### 3.1.2. Institutional Commitments

The State Committee on Food and Nutrition (SCFN) was established in both Kebbi and Anambra states, and it was led by the Permanent Secretary of Budget and Economic Planning, with the State Nutrition Officer acting as the committee’s secretary. While the SCFN in Kebbi had been active in the past, it had recently been inactive, with one participant suggesting that it was better described as “slumbering”. This was due to a significant decline in financial support from the United Nation’s Children Fund (UNICEF), which resulted in fewer meetings. As a solution, there were proposals to revive meetings and finance them from the state budget instead of relying on UNICEF.

In contrast, the SCFN in Anambra remained active and engaged, with regular meetings to finalize their state plan of action for nutrition. The participant attributed this to the involvement of UNICEF, which had played a significant role in pushing for meetings and developing plans. However, the closure of UNICEF’s office in Enugu temporarily halted activities until efforts resumed towards the end of the previous year, with the support of the new Permanent Secretary.

Regarding strategic nutrition plans of action, Kebbi State and some of its Local Government Areas (LGAs) had already finalized and published their plans in 2016. However, Anambra State and its LGAs still needed a plan of action, indicating different stages of progress between the two states.

#### 3.1.3. Budgetary Commitments

In 2018, both Kebbi and Anambra states had allocated budget provisions for nutrition-related interventions. Kebbi had explicitly allocated funds for nutrition, while Anambra’s budget provisions were for the SCFN functions. The participants from Kebbi expressed satisfaction with the adequacy of resources, while those from Anambra considered the resources substantial but inadequate. The allocation of resources was based on the needs and was influenced by the Governor, with the Commissioner of Health (The Commissioner of any Ministry (Health, Agriculture, etc.) is appointed by the State Governor to oversee, direct, and manage the Ministry in the state) having some influence in Anambra.

Both states considered food and nutrition programs to be adequately resourced. The Federal Government of Nigeria’s agricultural promotion policy [23] had an impact at the state level, with stand-alone agricultural programs for food production receiving sufficient resources. A participant from one state supported this view, highlighting the government’s focus on agriculture and successful initiatives such as the anchor borrowers’ program and rice programs.

The Governor of Anambra State strongly emphasized agriculture, but some feel that this focus was limited to Anambra East and West LGAs with other areas of the state being neglected. The health, agriculture, and education sectors were given the highest funding priority, while social welfare and the environment received comparatively less. Although there was a commitment to combat malnutrition and promote nutrition-sensitive approaches, budgetary limitations and limited institutional commitment constrained these efforts. For example, there was a decline in funding for ready-to-use therapeutic food in Kebbi, which was affected by budget constraints [8]. The budget sets specific priorities on the government’s agenda, with agricultural interventions receiving significant allocations due to the need for the government’s counterpart contribution to donor-initiated programs.

### 3.2. Agenda Setting: Opportunities to Advance Nutrition in the States

#### 3.2.1. Problem Stream

The problem stream revealed that malnutrition indicators were present in both Kebbi and Anambra states. Officials in Kebbi were aware of the statistics, and one participant expressed the desire to reduce the high malnutrition rates in the state. However, participants in Anambra showed disbelief and found it hard to accept malnutrition among children in their state.

A gap in local media coverage of nutrition issues was identified in both states. More qualitative and quantitative evidence should be presented on television or radio to raise public awareness. Participants emphasized the need for media involvement, stating that increased awareness of malnutrition prevalence would prompt action.

In Kebbi State, local radio played short songs and hosted talks on malnutrition. The biannual Maternal, Newborn, and Child Health Week (MNCHW) garnered significant attention, and visits by donor representatives to the State Executive Governor increased awareness. In Anambra State, stakeholders remained unaware of the extent of malnutrition prevalence. WHO standards classified the state’s stunting rate as “medium severity” at 18.4% [24]. The absence of active development partners, such as the closed UNICEF office in the Southeast region, further hindered progress.

The advocacy to address malnutrition in Anambra and Kebbi states relied on quantitative and qualitative evidence. Qualitative evidence significantly highlighted the issue in Anambra, but there were concerns about the state’s image. Both states had policy champions and advocacy activities. Still, Anambra needed more significant civil society influence for policy change, while Kebbi had traditional rulers like the Emir of Argungu, who held significant sway.

The campaigns, speeches, and influence of the “First Ladies” impacted both states. Although there was agreement in the advocacy community on the prevalence and causes of malnutrition, there were divergent opinions on the appropriate solutions. Some advocated for Infant and Young Child Feeding, while others highlighted the role of Ready-to-Use Therapeutic Food (RUTF).

Several factors influenced the prioritization of malnutrition. Kebbi had credible stunting indicators, while Anambra lacked such data. The availability and importance of indicators played a crucial role. In Anambra, reframing the issue to focus on impact rather than prevalence was necessary. Generating political commitment required establishing malnutrition as a problem and framing it according to WHO severity classification. Media representation and repeated short-term messages were essential for a compelling nutrition narrative.

Donors played a crucial role in maintaining the government’s focus on malnutrition in Kebbi State, frequently visiting and implementing programs. It was important to recognize the potential contributions of nutrition-sensitive sectors, as focusing solely on nutrition-specific interventions might overlook valuable opportunities. Incomplete policy solutions or failure could result in declining attention.

#### 3.2.2. Policy Stream

Apparent policy alternatives, including costed plans of action, were evaluated in both states. Both Anambra and Kebbi had detailed state nutrition strategies. In Anambra State, the costed plan of action was being produced during the research. The existence of a strategic plan of action led participants to agree that policy advocates were cohesive.

In Anambra State, participants from the Ministry of Environment argued for their inclusion in the State Committee on Food and Nutrition (SCFN) due to the impact of water, sanitation, and hygiene (WASH) on reducing malnutrition [25].

The study highlighted the key players who were instrumental in promoting nutrition policies in the states of Anambra and Kebbi. In Anambra, the permanent secretary of Budget and Planning took the initiative to revitalize the State Committee on Food and Nutrition (SCFN), while foreign development partners influenced the release of nutrition funds through the Governor and Commissioners in Kebbi. The policy entrepreneurs in Anambra were the First Lady and Commissioner of Health, while Kebbi State was the Ministry of Health. The donors were also recognized as policy entrepreneurs for exerting pressure on the government. Anambra State effectively utilized the SCFN as a policy platform, ensuring cohesion among policy advocates, while the inactive SCFN in Kebbi State led to an incohesive situation.

#### 3.2.3. Political Stream

In Kebbi State, the opportunity to make a political commitment to nutrition was more significant due to the scheduled legislative and executive elections in 2019. On the other hand, only legislative elections took place in Anambra State. Kebbi State received substantial technical and financial support from development partners and international agencies, while Anambra State received limited support.

The Governor of Kebbi State, previously a federal senator, showed commitment to education and expressed willingness to prioritize investment in education if funding became available. In both states, nutrition could be prioritized by leveraging the focus on agriculture and including nutrition within the food production objective. Maternal and child health was of particular interest in Kebbi State.

The political stream indirectly influenced the focus on malnutrition and its prevalence, shaped by national or state-level mood, social climate, public opinion, and political insecurity. However, a weak national mood hindered advocates and policy entrepreneurs from addressing the issue, acting as the highest inhibitor of the policy process [8]. During the data collection period (2017/2018), political insecurity and tensions arising from alleged clashes between herders, Boko Haram, and Biafra agitators dampened the national mood [26,27,28].

Based on the analysis of three data sources, it has been revealed that nutrition has become a significant policy priority. This issue is increasingly being recognized on state agendas, especially in Kebbi. Nigeria’s current narrative lacks a nutrition-sensitive framing, which calls for exploring sustainable and effective solutions to tackle this problem. To gain a better understanding, Table 2 presents a combined display of stakeholder qualitative and quantitative analysis.

### 3.3. Stakeholder and Institutional Analysis

The stakeholder analysis has identified strong supporters of nutrition in Kebbi, including the Budget and Economic Planning, Health, and Education Ministries, as well as the APC (All Progressives Congress), UNICEF, DFID (Department for International Development), and the EU (European Union). In Anambra, the Social Welfare Ministry was strongly supportive, while the Environment Ministry was neutral in both states due to limited SCFN involvement. In Kebbi, the Budget and Health Ministries, the APC, DFID, and EU had a high ability to advance nutrition policy, while all supporters except the Social Welfare Ministry had a high ability in Anambra.

The Budget and Planning, Health, Agriculture, and Education Ministries supported nutrition policies in both states. The Environment Ministry’s support varied, with Kebbi reporting moderate support and Anambra neutral. The Ministry of Social Welfare had strong support in Kebbi but moderate in Anambra. The moderate ability of the Social Welfare Ministry in Anambra to advance nutrition-related policies can be attributed to its limited resources and narrower focus on social welfare issues. Unlike ministries directly involved in health or agriculture, the Social Welfare Ministry may not have the same expertise, funding, or mandate to tackle nutrition policies effectively, impacting its overall influence in this domain. Political party support varied, with the All Progressive Grand Alliance (APGA) in Anambra strongly supporting nutrition activities.

Kebbi differed in two aspects: the Governor was APC, while the predecessor was People’s Democratic Party (PDP). Both strongly supported nutrition policy, but funding was higher previously. The recession impacted national revenue and state allocations. Political parties could not advance or block policy due to the lack of explicit ideologies. The Social Welfare and Environment Ministries could not advance nutrition policy [29]. In both states, foreign partners and United Nations (UN) bodies supported nutrition at different levels. UNICEF, DFID, and the EU supported nutrition in Kebbi and had a high ability to advance nutrition policy. Anambra had minimal presence.

## 4. Discussion

### 4.1. Policy Implications

Regarding political commitment and prioritization of food and nutrition policy, Kebbi State has shown a greater budgetary commitment towards nutrition-specific policies than Anambra State. However, Kebbi State’s Stronger SCFN has faced a decline in the number of meetings held due to changes in its membership and lower funding from UNICEF in the past year. To promote the nutrition objectives, it is essential to strengthen institutional commitment. It is difficult to distinguish between donor and government activities regarding nutrition programs and policing in Kebbi State due to the prominence of donor funding. Donors tend to work where commitment to the cause exists, intending to maximize progress and impact [30]. Governments are likely to request matching funds from donors. As a result, donors usually provide the majority of the funding and utilize their personnel to execute policies. However, if the goals of the government and donors are not aligned, discrepancies can occur during the development of policies or discussions about programs [31]. On the other hand, the government contributes a portion of the total resources but takes credit for such efforts.

Kebbi and Anambra states face challenges regarding problems, policy, and politics. However, there are also positive aspects to these issues. In Kebbi, for instance, malnutrition in children under five is recognized as a significant problem. This presents an opportunity for the media to improve reporting and raise awareness among the public. While ministerial responsibilities have been agreed upon in the policy stream, more cohesion is needed in advocacy efforts. This is in contrast to other regions where there’s a need for more cohesion in the policy community. Effective advocacy in establishing malnutrition as a problem would help in the fight against malnutrition in the state. Pelletier et al. [32] found that effective advocacy must consistently raise attention toward nutrition. Existing attention is driven by external resource provision. No ministry was categorized as opposing nutrition.

The findings align with other governance reports on Kebbi State, which show that the budgeted funds sometimes differ from the released funds. Although the SCFN and local governments were functional, they did not hold quarterly meetings. Unfortunately, the Civil Society Organization had no active engagement in the nutrition work. The state government typically receives its allocation from federal funds, but the spending decisions are made at the state governor’s office. While no stakeholder group has opposed the nutrition agenda, the insufficient expenditure allocations indicate that the state still needs to prioritize nutrition [14].

Despite many policies and plans, government coordination and implementation could be more robust. This weak implementation has been reported in other sectors in Nigeria [33,34]. Government partners (UNICEF, DFID, USAID) and NGOs lead the nutrition policy and programming environment.

Anambra State is committed to addressing the issue of malnutrition, but its budget allocation does not reflect this commitment. The state’s malnutrition profile could worsen if adequate attention is not given to nutrition. While programme managers prioritize nutrition, their commitment may be more rhetorical than actual. The low budget allocation may be due to delayed release of funds rather than unavailability.

Nutrition appears to have low priority in the Anambra state, although the policy stream is strengthening in line with the current development of the state nutrition plan. If not backed with action stemming from the awareness of nutrition problems, this plan might be hard to implement. Policymakers are more likely to be influenced by the extent of the problem than by a coherent, evidence-based policy [32]. Generating interest without the help of foreign partners or donors is crucial for stakeholders in this state. Despite the recent re-election of the Governor, which offers limited opportunity for political support, interest can be generated through the legislative route. Ministries that actively support nutrition have faced budget constraints but still expressed their commitment. However, they need to be reminded of the nutrition-sensitive nature of their policies.

### 4.2. Implications of Political Will on Malnutrition and Nutrition-Sensitive Programming

Based on the analysis, it is evident that different strategies are required to obtain different forms of commitment from the states. The Governor is responsible for budgetary commitment, particularly for funding nutrition programs. Therefore, advocacy aimed at the Governor and commissioners/permanent secretaries is the most effective way to gain support. Senior government officials hold institutional commitment and have the authority to institutionalize nutrition by creating nutrition desks and assigning responsibilities. Moreover, they play a vital role in developing the annual budget. Mid-level, senior government officials or offices of the Governor and First Lady usually express commitments to nutrition. The mid-level staff has a role in ensuring that institutions and the programmes they design are implemented adequately. Both states lack horizontal integration of all ministries, specifically, a programmatic linkage that ties all ministries together [32]. This integration is needed to establish nutrition-sensitive practices from programme design to implementation in the field.

Table 3 presents a SWOT analysis for Kebbi and Anambra states, revealing varied dynamics in their approaches to nutrition. Kebbi, with a high stunting prevalence of 60.6%, shows a strong political commitment to nutrition yet faces challenges like high dependency on donor funds and an inactive SCFN. Opportunities lie in integrating nutrition with other sectors and leveraging advocacy platforms, while threats include the potential withdrawal of donor support and a narrow focus on nutrition-specific interventions.

Anambra, with a lower stunting prevalence of 18.4%, benefits from an active SCFN. However, the perception that nutrition is not a significant problem, coupled with minimal media attention, poses a challenge. Opportunities for Anambra include stronger integration of nutrition with agriculture and targeted advocacy, but it faces threats from denying malnutrition as a critical issue.

The strategies emerging from this matrix offer pathways for both states to enhance their nutrition policies. For Kebbi, this involves strengthening sectoral integration and diversifying funding sources, while Anambra should focus on elevating the importance of nutrition in public and political agendas and leveraging agricultural integration. These strategic directions address each state’s specific contexts and align with broader national efforts to combat malnutrition.

### 4.3. Strengths and Limitations of the Study

A key strength of this research is its contextually bound case study. Including the contextual boundaries means the case is adequately defined [35]. Additional information, such as the institutional, political, and other contexts, improves case understanding [35].

The study recognizes several limitations, including the subjectiveness of the qualitative approach, which can result in overly optimistic or pessimistic views depending on the complexity of the issues [36]. Despite this, the qualitative approach provides a deeper understanding of complex problems. The assessment was limited to the alignment of nutrition-sensitive policies, and not all potential interviewees agreed to participate, leading to a possibility of bias in the results. However, integrating findings from interviews and workshops presented reliable data on the political will in both states [37].

There is also the challenge of eliciting unbiased data from the study participants, who also happen to be government officials. Given the complexity of this topic and that they were required to assess the performance of their employer, some bias was expected.

The qualitative methods applied in this study yielded several key outputs. These included detailed insights into the current levels of political commitment to nutrition-sensitive interventions in Kebbi and Anambra States, an understanding of recent shifts in policy priorities, and identifying strategic opportunities for advancing food and nutrition policies. Furthermore, the qualitative approach facilitated the development of tailored strategies to enhance political backing for these policies within the specific political contexts of the two states.

## 5. Conclusions

This study aimed to investigate the role of political commitment, windows of opportunity, and stakeholder analysis in nutrition programming in two Nigerian states—Kebbi and Anambra. The study found that the severity of malnutrition and the recognition of government officials greatly influence the level of political commitment. It was also found that the involvement of multiple stakeholders is crucial for effective nutrition programming and that strengthening existing coordination committees is essential. Policy windows exist for integrating nutrition outcomes across sectors, leveraging local media for strategic framing and advocacy. Understanding the political landscape is important for nutrition stakeholders to garner support and advocate for nutrition issues. The study offers a practical method to explore political commitment in other states and countries, and the PCOM RAT can help facilitate future comparisons across Nigerian states. Future research could expand on this study by examining the long-term impacts of political commitment and stakeholder involvement on nutrition outcomes in these regions. Comparative studies across more Nigerian states or other countries with similar socio-political environments could provide deeper insights into the effectiveness of different policy approaches and stakeholder strategies in addressing malnutrition. Additionally, investigating the role of media and communication strategies in shaping public perception and political action on nutrition issues presents another promising avenue for further exploration.

## Figures and Tables

**Table 1:** Workshop participants.

	Agriculture	Education	Environment	Water	Welfare	Health	Total
Anambra State	1	3	3	0	2	0	9
Kebbi State	0	2	1	2	1	3	9

**Table 2:** Joint display of political commitments: subscale description and comparison.

Domain		Radar Point	Stakeholder’s Experience (Evidence from Workshop Notes)	Stakeholder’s Experience (Evidence from the Key Informant Interviews)	Strategies for Generating Political Commitment
Prioritization of nutrition					
Expressed commitment.	Kebbi	85.7 percent.	Speeches of the Director of Child Development, Ministry of Social Welfare. Inauguration of boreholes in the Water Ministry.	MNCHW. Medicaid rally.	Stated commitment from not only the Governor and his delegates but also the commissioners and the permanent secretaries of the various nutrition-sensitive sectors. Especially agriculture and education.
	Anambra	71.4 percent.	MNCHW. Ministry of Education Speeches.	MNCHW. Any activity of the Ministry of Health. The state utilizes all opportunities to present all aspects of health, given that funding is limited.	Same as above. Especially agriculture and social welfare.
Institutional commitment.	Kebbi	66.7 percent.	Inactive SCFN.	Slumbered SCFN	Reviving the SCFN. Budgeting for the SCFN to ensure meetings are held irrespective of donor funding.
	Anambra	66.7 percent.	Functional SCFN.	Functional SCFN	Sustaining current momentum. Ensure nutrition awareness steps down from ministry heads and representatives at the SCFN to the implementers and other actors.
Budgetary commitment.	Kebbi	75 percent.	Funding is available, though nothing in the budget can be funded. Funding support from the donor partners keeps the projects going.	Commitment changes. RUTF was funded last year, and this year, another focus has been chosen.	Sustained budgetary commitment irrespective of donor funds.
	Anambra	33.3 percent.	Average funding available.	Funding is mostly late. The Commissioner of the Health Ministry could push for funding to be released through “memos” to the Governor.	Getting the buy-in of the Governor, the biggest veto power for funding.
Policy window of opportunity					
Problem stream.	Kebbi	80 percent.	No media awareness. No visible civil society. Traditional rulers as influencers. A high-powered panel of foreign donors held a workshop for the Governor is the most significant focusing event.	The high stunting prevalence in Kebbi State was a concern to stakeholders. Few Civil Society Organizations on the ground, but disconnected and inconsistent.	Mobilizing local media houses to pay attention to nutrition. Sustain the current awareness of the high malnutrition prevalence in that state.
	Anambra	60 percent.	No media awareness. The SCFN chairman is the primary influencer. Visit of SCFN to the field (schools and rural areas) is the focusing event for nutrition.	The marginally lower stunting rate in the state has clouded the severity of stunting in the state. The CSOs are based in malnutrition hotspots but lack the power to influence policy.	Establishing that despite low malnutrition prevalence in the state, efforts need to be on the ground to eliminate malnutrition and prevent increasing overweight/obesity. Mobilizing local media houses to pay attention to nutrition. Coordination of the grassroots CSO to be able to influence policy.
Policy stream.	Kebbi	75 percent.	Published strategic plan of action for nutrition. No single individual influences policies—that onus goes to the foreign donors.	Non-cohesiveness among policy advocates concerning solutions for acute malnutrition. Ministry of Health as a policy entrepreneur.	
	Anambra	100 percent.	The strategic plan of action ready to be printed and then launched. The chairman of the SCFN is the main driver of nutrition policies.	The First Lady and the Health Commissioner are policy entrepreneurs for nutrition.	
Politics.	Kebbi	100 percent.	The open window during the 2019 elections. Massive funding from external donors. Governments’ priority is education.	Maternal and child health as a government priority.	Advancing nutrition-sensitive education policies in the state. Policy advocacy to the state legislators on narratives to advance nutrition in the state.
	Anambra	50 percent.	A minimal open window in the 2019 legislative elections and the current Governor’s 2nd term swearing-in. Very minimal funding from foreign agencies. Government’s priority is agriculture.	Agriculture as a government priority.	Advancing nutrition-sensitive agricultural programmes in the state. Policy advocacy to state legislators on narratives to advance nutrition in the state.
Stakeholders/Institutional Analysis					
Interest group mobilization.	Kebbi	100 percent.	Strong supporters include APC (All Progressives Congress), UNICEF (United Nations Children’s Fund), DFID (Department of International Development, now called Foreign, Commonwealth, and Development Office (FCDO)), EU (European Union), Budget, Health, Agriculture and Education ministries. The Ministry of Social Welfare is a moderate supporter. The Ministry of Environment is a neutral party.	Ministry of Environment categorized as supporting nutrition moderately. The past government might have been better disposed to nutrition, though that government had a higher allocation from the Federal Government.	Addition of the Ministry of Environment as a member of the SCFN from the federal level down to state and LGA. Sustained donor activity.
	Anambra	100 percent.	Strong supporters include Ministries of Budget, Health, Agriculture, Education, and Health. Ministry of Environment as a neutral party. UNICEF provides moderate support.	Ministry of Environment is a neutral party with regard to nutrition in the state. Ministry of Social Welfare provided moderate support for nutrition in the state.	Addition of the Ministry of Environment as a member of the SCFN from the federal level down to state and LGA. A return of UNICEF region to the South East.

**Table 3:** SWOT analysis of the political economy in Kebbi and Anambra states, Nigeria.

	Stunting Prevalence	Strengths	Weakness	Opportunities	Threats
Kebbi	60.6 percent.	High level of attention paid to nutrition in the current administration.	High dependence on donor agencies. Inactive state Committee for Food and Nutrition. Very little media attention on malnutrition.	Pairing nutrition firmly with nutrition-sensitive sectors. Employing the First Lady as an active advocate.	Withdrawal of donor funds. Focus on only nutrition-specific interventions as the pathway to adequate nutrition.
Anambra	18.4 percent.	Active state Committee for Food and Nutrition.	Nutrition not viewed as a problem. Very little media attention on malnutrition.	Pairing nutrition firmly with nutrition-sensitive sectors with agriculture. Advocacy to the Governor on nutrition.	Persistent denial of malnutrition as a prominent issue in the state.

## Data Availability

Raw data, both print and electronic, and the dissertation are being stored at the Division of Human Nutrition at Stellenbosch University. The data can be made available by request to the corresponding author.
